# Determinants of fibrotic atrial cardiomyopathy in atrial fibrillation. A multicenter observational study of the RETAC (reseau européen de traîtement d’arrhythmies cardiaques)-group

**DOI:** 10.1007/s00392-021-01973-1

**Published:** 2021-12-02

**Authors:** Björn Müller-Edenborn, Zoraida Moreno-Weidmann, Sandrine Venier, Pascale Defaye, Chan-il Park, José Guerra, Concepcion Alonso-Martín, Victor Bazan, Xavier Vinolas, Enrique Rodriguez-Font, Bieito Campos Garcia, Serge Boveda, Stéphane Combes, Jean-Paul Albenque, Benoit Guy-Moyat, Dietmar Trenk, Martin Eichenlaub, Juan Chen, Heiko Lehrmann, Franz-Josef Neumann, Thomas Arentz, Amir Jadidi

**Affiliations:** 1 Department of Cardiology and Angiology II, Section for Electrophysiology, Heart Center, University of Freiburg, Südring 15, 79189 Bad Krozingen, Germany; 2 Department of Cardiology, Julius-Hospital, Würzburg, Germany; 3 Department of Electrophysiology, Hospital de la Santa Creu i Sant Pau, Universitat Autonoma de Barcelona, CIBERCV, Barcelona, Spain; 4 Department of Cardiology, University Hospital Grenoble, Grenoble, France; 5 Department of Cardiology, Clinique de la Tour, Geneva, Switzerland; 6 Heart Rhythm Management Department, Clinique Pasteur, Toulouse, France; 7 Department of Cardiology, University Hospital Limoges, Limoges, France; 8 Department of Cardiology and Angiology II, Section for Pharmacology, Heart Center, University of Freiburg, Bad Krozingen, Germany; 9 Department of Electrophysiology, University Hospital Mainz, Mainz, Germany; 10 Department of Cardiology and Angiology II, Heart Center, University of Freiburg, Bad Krozingen, Germany

**Keywords:** Atrial fibrillation, Risk stratification, Pulmonary vein isolation, Fibrotic atrial myopathy

## Abstract

**Aims:**

Despite advances in interventional treatment strategies, atrial fibrillation (AF) remains associated with significant morbidity and mortality. Fibrotic atrial myopathy (FAM) is a main factor for adverse outcomes of AF-ablation, but complex to diagnose using current methods. We aimed to derive a scoring system based entirely on easily available clinical parameters to predict FAM and ablation-success in everyday care.

**Methods:**

In this multicenter, prospective study, a new risk stratification model termed AF-SCORE was derived in 220 patients undergoing high-density left-atrial(LA) voltage-mapping to quantify FAM. AF-SCORE was validated for FAM in an external mapping-validation cohort (*n* = 220) and for success following pulmonary vein isolation (PVI)-only (without adjunctive left- or right atrial ablations) in an external outcome-validation cohort (*n* = 518).

**Results:**

FAM was rare in patients < 60 years (5.4%), but increased with ageing and affected 40.4% (59/146) of patients ≥ 60 years. Sex and AF-phenotype had additional predictive value in older patients and remained associated with FAM in multivariate models (odds ratio [OR] 6.194, *p* < 0.0001 for ≥ 60 years; OR 2.863, *p* < 0.0001 for female sex; OR 41.309, *p* < 0.0001 for AF-persistency). Additional clinical or diagnostic variables did not improve the model. AF-SCORE (+ 1 point for age ≥ 60 years and additional points for female sex [+ 1] and AF-persistency [+ 2]) showed good discrimination to detect FAM (c-statistic 0.792) and predicted arrhythmia-freedom following PVI (74.3%, 54.7% and 45.5% for AF-SCORE ≤ 2, 3 and 4, respectively, and hazard ratio [HR] 1.994 for AF-SCORE = 3 and HR 2.866 for AF-SCORE = 4, *p* < 0.001).

**Conclusions:**

Age, sex and AF-phenotype are the main determinants for the development of FAM. A low AF-SCORE ≤ 2 is found in paroxysmal AF-patients of any age and younger patients with persistent AF irrespective of sex, and associated with favorable outcomes of PVI-only. Freedom from arrhythmia remains unsatisfactory with AF-SCORE ≥ 3 as found in older patients, particularly females, with persistent AF, and future studies investigating adjunctive atrial ablations to PVI-only should focus on these groups of patients.

**Graphical abstract:**

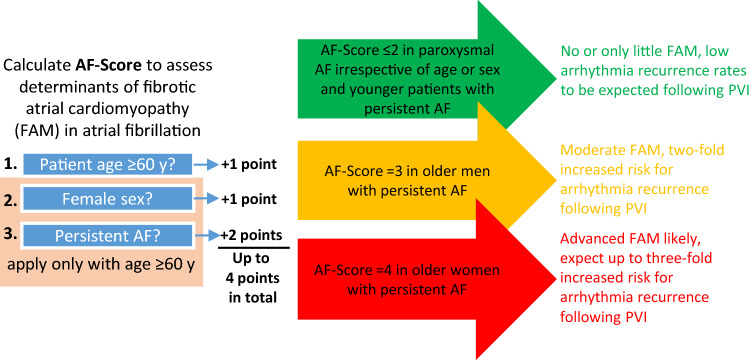

**Supplementary Information:**

The online version contains supplementary material available at 10.1007/s00392-021-01973-1.

## Introduction

Pulmonary vein isolation (PVI) is the mainstay of therapy for the interventional treatment of atrial fibrillation (AF), and arrhythmia freedom can be achieved in up to 80% of patients [1, 2].

In fibrotic atrial myopathy (FAM), areas of atrial fibrosis promote heterogeneous conduction that can itself perpetuate AF even in the absence of pulmonary-vein inputs [3]. As a result, FAM is associated with adverse outcomes following PVI, and arrhythmia recurrences occur in up to 50% of patients with FAM [4–7].

To select the most appropriate therapeutic strategy in patients with AF, a personalized approach based on the presence or absence of FAM would be desirable. While FAM can be diagnosed non-invasively using contrast-enhanced cardiac magnet resonance imaging or various ECG-parameters, their widespread use for screening-purposes is significantly limited by inherent methodological restrictions and availability.

This is particularly true in the primary care-setting in which the vast majority of patients with AF are taken care for. As a result, several risk stratification models were proposed to identify patients-at-risk for FAM. However, while offering moderate to good diagnostic properties, these models still require diagnostic tests such as echocardiography or blood sampling.

For the current study, we hypothesized that a condensed risk model based entirely on descriptive patient information available through simple medical history taking may yield comparable power to predict FAM and ablation-outcome. In addition, such risk model may allow a better understanding of contemporary trials on AF-ablation, as such descriptive variables are likely available in the published study results.

## Methods

### Study design and patient populations

In this multicenter prospective observational study, consecutive patients undergoing first PVI between January 2016 and October 2019 at the participating sites (University Heart Center Freiburg-Bad Krozingen, Bad Krozingen, Germany; Clinique Pasteur, Toulouse, Centre Hospitalier Universitaire Limoges, Limoges, and Centre Hospitalier Universitaire Grenoble, all in France; Hopital de la Tour, Geneva, Switzerland, and Hopital de Sant Pau, Barcelona, Spain) were screened for inclusion into the determination cohort and underwent high-density endocardial mapping as outlined below. An outcome-database of patients who underwent their first PVI at University Heart Center Freiburg-Bad Krozingen and Centre Hospitalier Universitaire Grenoble served as external outcome-validation cohort (Supplemental Fig. 1). Details on ablation and follow-up are described below. Inclusion criteria were symptomatic paroxysmal (< 7 days duration) and persistent (> 7 days and < 12 months duration) atrial fibrillation. Exclusion criteria were prior left atrial ablation, presence of left atrial thrombus or contraindication to anticoagulant therapy. Primary endpoints were the derivation of a scoring system to predict left-atrial low voltage-substrate as a surrogate of FAM, and external validation of this score for prediction of FAM. Secondary endpoint was the external validation of this score to predict arrhythmia-freedom following PVI.

### High-density mapping of the determination cohort

Patients in the determination cohort underwent high density left-atrial voltage mapping during sinus rhythm with a minimum of 1200 mapped points per patient [8]. All patients underwent mapping in sinus rhythm prior to any ablation using an endocardial electro-anatomical contact mapping-system (Carto3, Biosense-Webster, Diamond Bar, CA, US) in combination with a 20-pole circumferential mapping catheter (electrode size: 1 mm; spacing: 2–6–2 mm). Peak-to-peak intracardiac bipolar electrograms were recorded at 15 to 250 Hz and amplified 0.1–0.2 mV/cm to visualize low-voltage electric activity. For highest accuracy, mapping was performed with respiratory gating and under mechanical ventilation. Low interpolation settings (17 in Carto3-system) were used. Mapping points that were > 5 mm away from the atrial geometry were excluded. Areas demonstrating potential low voltage when mapped with the 20-pole lasso catheter were reconfirmed using a contact force-enabled mapping catheter with a contact threshold of > 5 g. LVS was defined using a cutoff value for bipolar peak-to-peak voltage in sinus rhythm of < 0.5 mV and further expressed in cm^2^ of absolute cumulative left-atrial surface area. The pulmonary vein antrae and pulmonary veins that physiologically show voltages < 0.5 mV as well as the mitral valvular area were excluded. For the current study, FAM was defined as LVS exceeding 5 cm^2^.

### Ablation procedure and outcome estimation in the outcome-validation cohort

All patients of the outcome-validation cohort underwent proximal circumferential pulmonary vein isolation without adjunctive left- or right-atrial ablations using either contact-force enabled radiofrequency-ablation in combination with the Carto3-Mapping system (Biosense Webster) or cryo-balloon-ablation (Medtronic Arctic Front Advance). The decision per ablation type was at the discretion of the treating physician. Antiarrhythmic drugs, where applicable, were continued for three months following ablation and then stopped. Patients underwent routine ambulatory cardiological examinations including 12-lead ECG at four weeks following ablation. Regular 24 h-Holter-ECGs were scheduled in 6-months-intervals. Patients experiencing symptoms suggestive of arrhythmia recurrence (e.g. palpitations, dyspnea, fatigue) underwent additional cardiological examinations including ECG and symptom-triggered ECG (event recorder and 24 h-Holter ECG). AF or atrial tachycardia lasting for > 30 s experienced beyond the blanking period of three months were considered as recurrence. The study protocol was approved by the ethic committees of the participating sites.

### Statistical analysis

Statistical analysis was performed using SPSS 25.0 for Windows (IBM Corporation, Armonk, NY) or Graphpad Prism 8 for Windows (Graphpad Software, La Jolla, CA). Data were checked for normal distribution using the Shapiro–Wilk test. Continuous variables were compared between groups using t-test or non-parametric testing or One-way ANOVA with Bonferroni-post hoc correction depending on normality and number of groups. Categorical variables were analyzed using Fisher exact test. Uni- or multivariate logistic regression was used to estimate probability ratios between groups. Unadjusted and adjusted arrhythmia-freedom rates were calculated by cox regression analysis. A two-sided *p* ≤ 0.05 was considered significant in all tests.

## Results

### Patient characteristics associated with fibrotic atrial myopathy

In total, 440 patients were included in the determination cohort and underwent high-density left atrial endocardial voltage mapping. Patients of the determination cohort were randomized 1:1 to the derivation cohort or the mapping-validation cohort (*n* = 220 each, Supplemental Fig. 1). These cohorts did not differ with regard to key patient characteristics (Supplemental Table 1). Factors associated with FAM in the derivation cohort in univariate models were age, female sex, arterial hypertension, persistent AF, left atrial (LA) dilatation and creatinine clearance. In a multivariate logistic model, only age, sex and AF-phenotype remained as independent factors associated with FAM (Table 1).

**Table 1:** Uni- and multivariate logistic regression for FAM in the determination cohort

	All patients	No FAM	FAM	Regression analysis			
	*n* = 220	*n* = 157	*n* = 63	Unadjusted		Adjusted	
				OR (95% CI)	*p*	OR (95% CI)	*p*
Age (years)	61.1 (11.7)	58.2 (12.2)	68.3 (6.3)	1.130 (1.08–1.18)	< 0.0001		
Age > 60 years	146 (66.4)	87 (55.4)	59 (93.7)	11.868 (4.11–34.27)	< 0.0001	6.194 (1.94–19.8)	0.002
Female sex	60 (27.3)	34 (21.7)	26 (41.3)	2.542 (1.36–4.77)	0.004	2.863 (1.28–6.4)	0.011
Body mass index (kg/sqm)	28.0 (4.4)	27.9 (4.2)	28.2 (4.7)	1.018 (0.95–1.09)	0.598		
Arterial hypertension	139 (63.2)	89 (56.7)	50 (79.4)	2.939 (1.48–5.84)	0.002	1.147 (0.49–2.70)	0.753
Diabetes mellitus	19 (8.6)	11 (7.0)	8 (12.7)	1.931 (0.74–5.05)	0.180		
Coronary artery disease	37 (16.8)	23 (14.6)	14 (22.2)	1.665 (0.79–3.49)	0.177		
Persistent atrial fibrillation	144 (65.5)	82 (52.2)	62 (98.4)	56.71 (7.7–419.1)	< 0.0001	41.309 (5.34–319.48)	< 0.0001
LV dysfunction (LVEF < 45%)	17 (7.7)	10 (6.4)	7 (11.1)	1.837 (0.67–5.06)	0.239		
LVEDD (mm)	51.1 (4.4)	51.2 (4.5)	50.9 (4.3)	0.981 (0.92–1.05)	0.564		
LA dilatation (> 40 mm)	173 (78.6)	115 (73.2)	58 (92.1)	4.237 (1.59–11.28)	0.004	2.221 (0.69–7.15)	0.181
Creatinine clearance (ml/min/1.73 sqm)	78.3 (15.1)	80.1 (14.7)	73.8 (15.2)	0.972 (0.95–0.99)	0.006	0.997 (0.97–1.02)	0.789
Cumulative area < 0.5 mV (sqcm)	6.1 (12.0)	0.8 (1.3)	19.2 (16.0)	n/a			
cumulative area < 1.0 mV (sqcm)	15.2 (21.1)	5.0 (6.2)	40.5 (23.5)	n/a			

FAM, fibrotic atrial myopathy (prespecified as ≥ 5 cm^2^ with bipolar voltage < 0.5 mV); LA, left atrial; LV, left ventricle; LVEDD, left-ventricular end-diastolic diameter

Values are given as mean ± standard deviation or *n* (%)

### Age-related development of fibrotic atrial myopathy

**Fig. 1:**
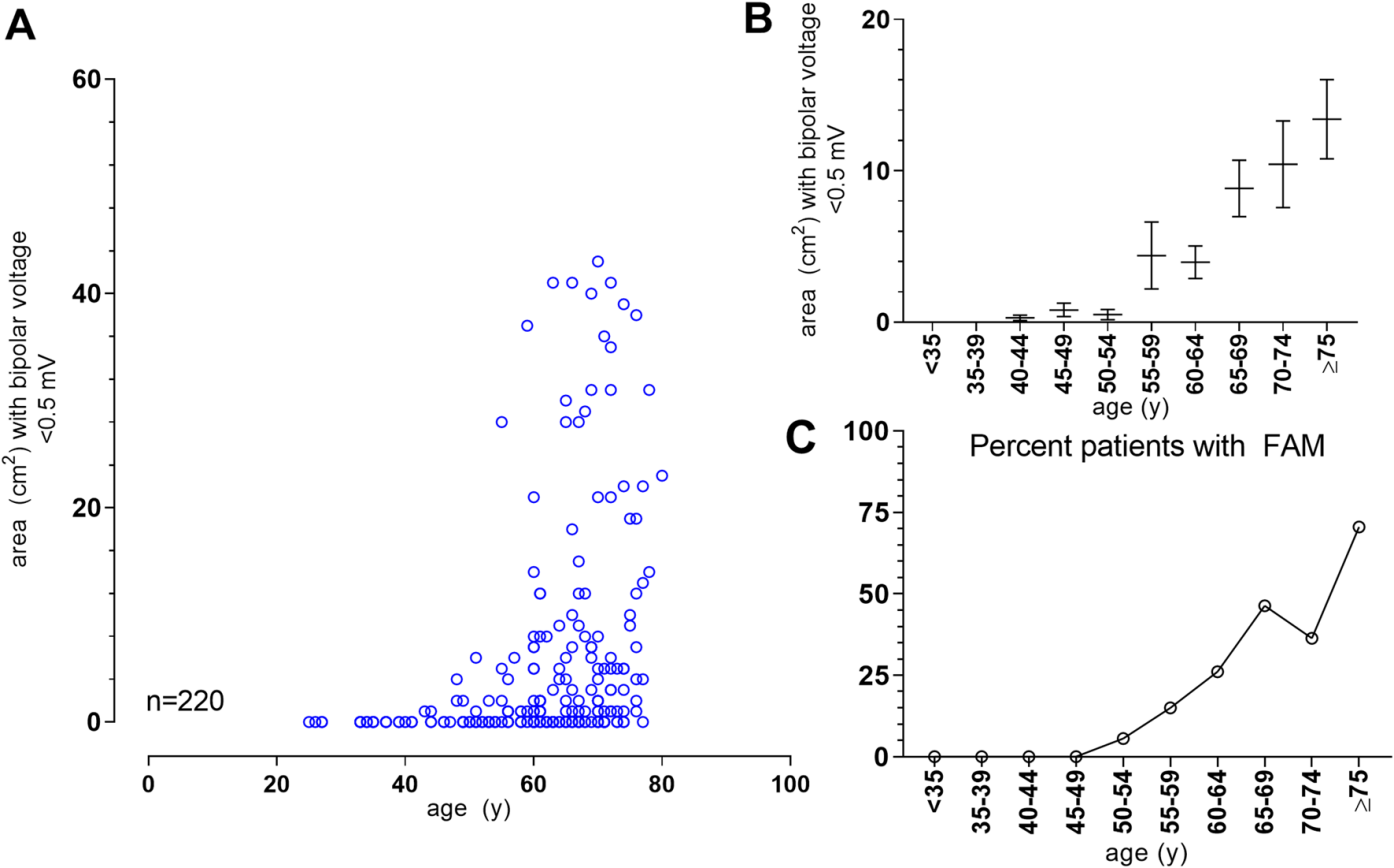
Age-related development of fibrotic atrial myopathy. Shown is the cumulative area with bipolar voltages < 0.5 mV per patient vs. patient age in the derivation cohort (**A**). Panel (**B**) gives the mean and standard error of cumulative areas with voltages < 0.5 mV in age-categories spanning 5 years each. The percentage of patients within each given age group meeting the prespecified criterion for FAM (≥ 5 cm^2^ low-voltage areas at < 0.5 mV) is shown in (**C**). FAM, fibrotic atrial myopathy

For the derivation cohort, the cumulative left-atrial area demonstrating bipolar voltages < 0.5 mV in relation to the patient’s age is given in Fig. 1. Patients who met the prespecified criterion for diagnosis of FAM (≥ 5 cm^2^ low-voltage areas at < 0.5 mV) had on average 19.2 ± 15.9 cm^2^ of cumulative low-voltage areas, compared to 0.8 ± 1.3 cm^2^ in patients who did not. Extensive low-voltage areas were primarily found in patients aged 60 years or older (8.4 ± 13.6 cm^2^ vs. 1.4 ± 5.4 cm^2^ in patients younger 60 years, *p* < 0.0001). The criterion for FAM (≥ 5 cm^2^ low-voltage areas at < 0.5 mV) was met in 59 of 146 patients (40.4%) aged 60 years or older, compared to only 4 of 70 (5.4%) in younger patients (*p* < 0.0001). The percentage of patients in a given age group with FAM increased steadily from 0% (0/38) in patients younger 44 years to 36% (31/87) in patients aged 60–69 years and reached 70% (12/17) in those aged 75 years or older (Fig. 1).

### Impact of clinical AF-phenotype on presence of fibrotic atrial myopathy

**Fig. 2:**
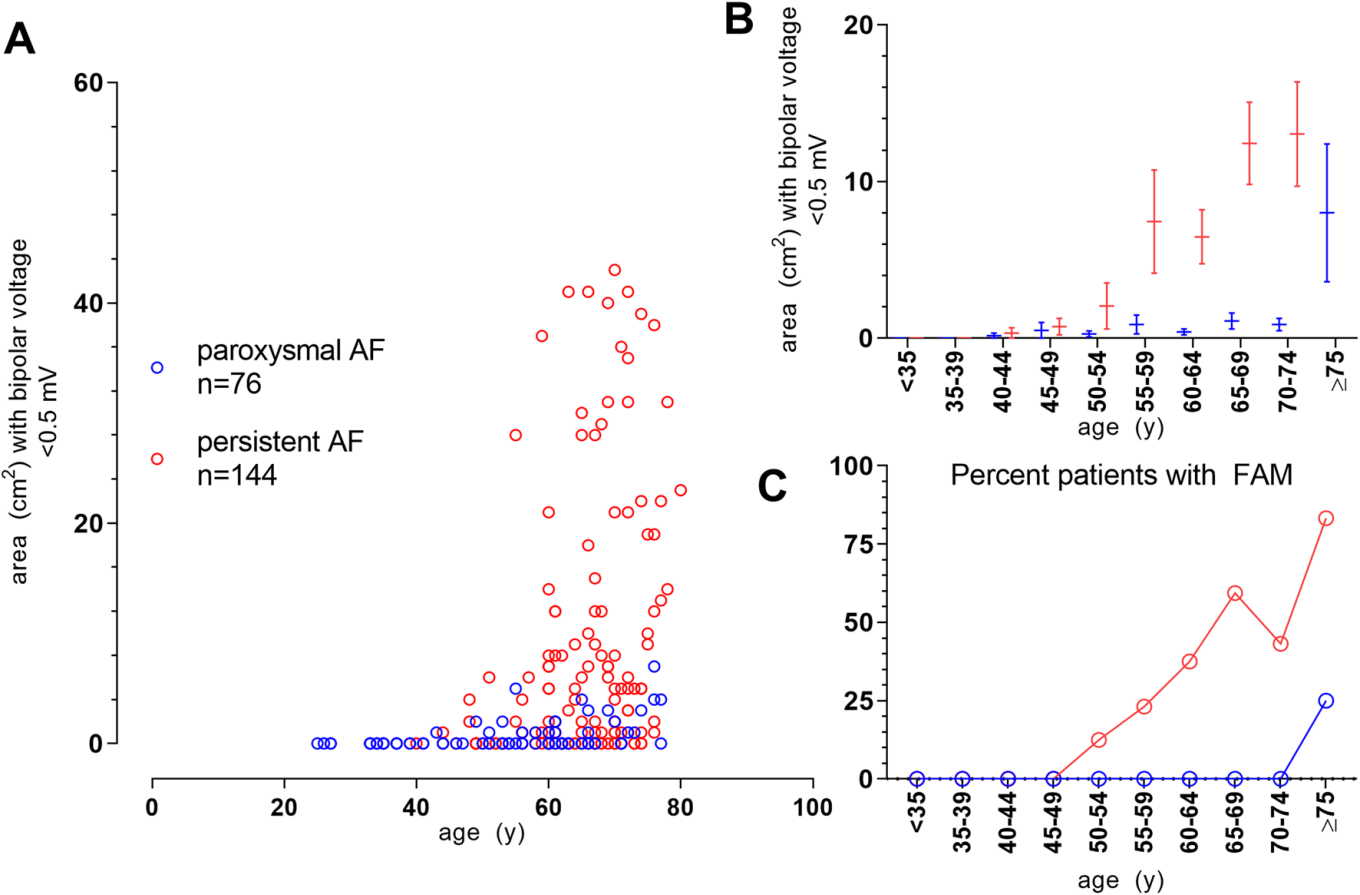
Clinical AF-phenotype and age-dependency of fibrotic atrial myopathy. Shown is the cumulative area with bipolar voltages < 0.5 mV per patient vs. patient age in the derivation cohort, grouped for paroxysmal AF (blue) or persistent AF (red, Panel **A**). Panel (**B**) gives the mean and standard error of cumulative areas with voltages < 0.5 mV in age-categories spanning 5 years each separated for paroxysmal AF (blue) or persistent AF (red). The percentage of patients within a given age group meeting the prespecified criterion for FAM (≥ 5 cm^2^ low-voltage areas at < 0.5 mV) in the respective groups is shown in (**C**). AF, atrial fibrillation; FAM, fibrotic atrial myopathy

Persistent AF with the longest recorded episode of AF exceeding 7 days was present in 42% (31/74) of younger and 77% (113/146) of patients aged 60 years or older (*p* < 0.0001). The presence of FAM increased incrementally with age in patients with persistent AF, reaching up to 84% in those aged 75 years or older (Fig. 2). Younger patients (< 60 years) rarely had FAM despite a clinical phenotype of persistent AF (4/31, 12.9%).

This observation was also made regarding the absolute extent of low-voltage areas, which were higher in persistent compared to paroxysmal AF only in patients older than 60 years (10.5 ± 14.8 cm^2^ vs. 1.2 ± 1.7 cm^2^ and 3.0 ± 8.1 cm^2^ vs. 0.3 ± 0.8 cm^2^ for persistent vs. paroxysmal AF in patients older and younger than 60 years, respectively; Supplemental Fig. 2).

Irrespective of age, FAM was rare in patients with paroxysmal AF and occurred in 1.3% (1/76) of cases only. The negative predictive value of a paroxysmal AF-phenotype for the absence of FAM was 100% in younger and 96.9% in patients aged 60 years and older. Irrespective of age, AF-persistency was highly sensitive, yet not specific, for FAM (Supplemental Fig. 2B).

### Female Sex and fibrotic atrial myopathy

**Fig. 3:**
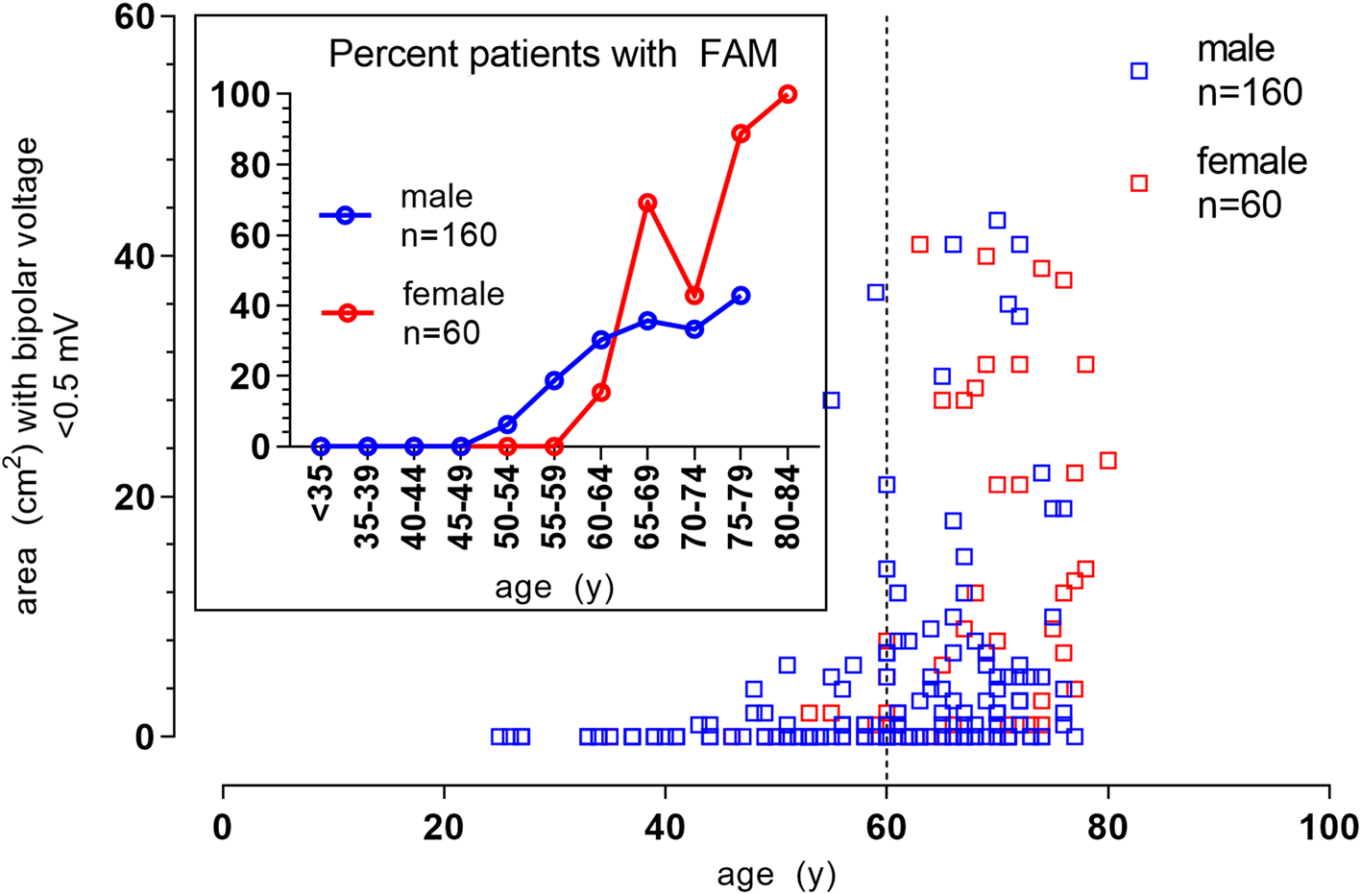
Impact of sex on fibrotic atrial myopathy. The cumulative area with voltages < 0.5 mV in individual patients in the derivation cohort vs. patient age and sex (females in red, males in blue) is shown in the large Panel. The inlay demonstrates the percentage of females and males meeting the prespecified criterion for FAM (≥ 5 cm^2^ low-voltage areas at < 0.5 mV)

**Fig. 4:**
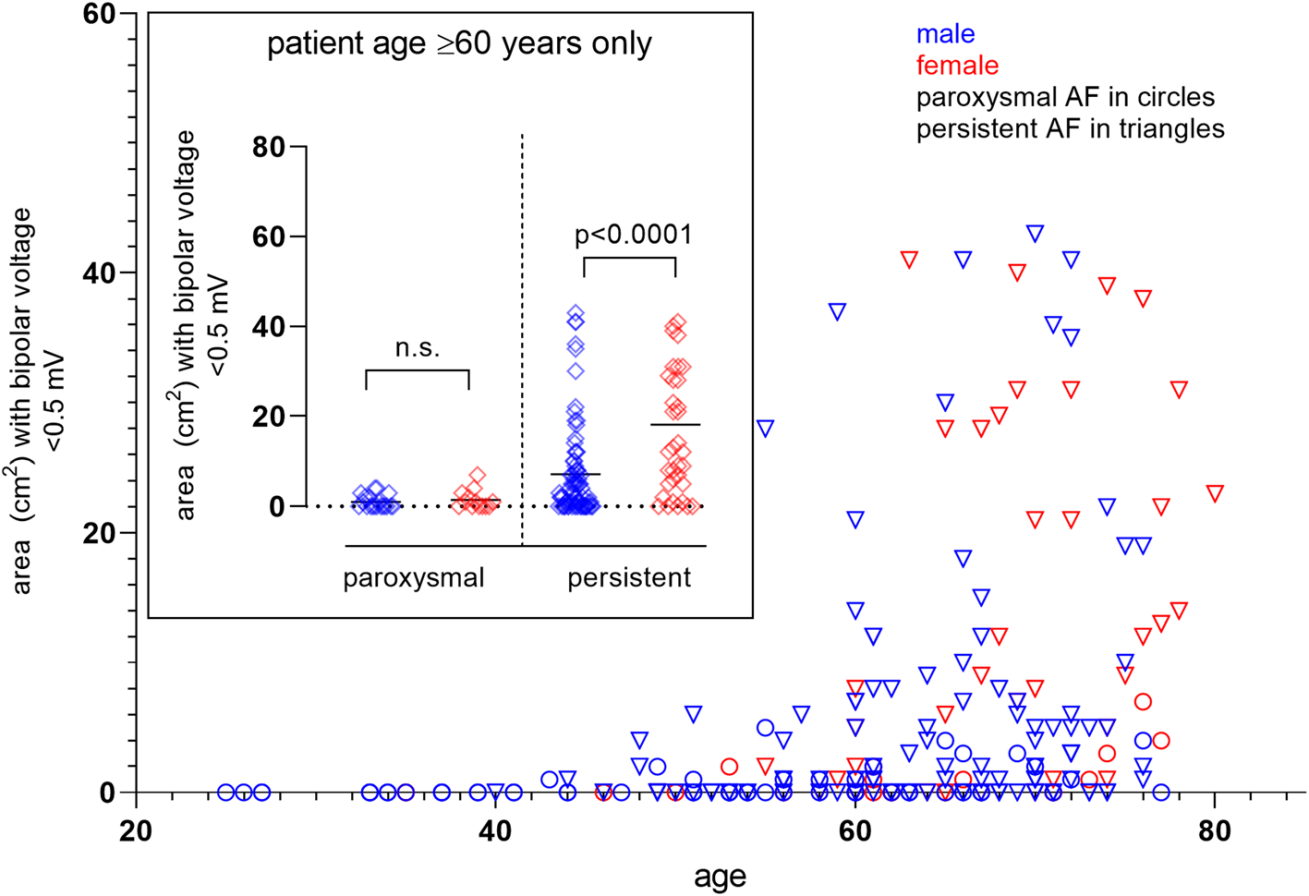
Sex and AF-phenotype determine fibrotic atrial myopathy in advanced age. The large panel shows the distribution of areas with bipolar voltages < 0.5 mV in the derivation cohort in women (red), men (blue) with paroxysmal (circles) and persistent AF (triangles). The small panel compares the extent of low-voltage areas between sexes and AF-phenotype restricted to patients aged ≥ 60 years. AF, atrial fibrillation

While the majority of study patients were male (160/220, 72.7%), women had FAM more often and more severe than men (26/60 females with FAM [43.3%] vs 37/160 males [23.1%], *p* < 0.0001; and low-voltage areas < 0.5 mV of 10.9 ± 17.2 cm^2^ in females vs. 4.2 ± 8.7 cm^2^ in males, *p* < 0.0001, Supplemental Fig. 3). The higher overall prevalence of FAM in females was driven by older women with persistent AF (Figs. 3 and 4; 18.1 ± 19.5 cm^2^ in females with persistent AF aged ≥ 60 years vs. 7.1 ± 10.5 cm^2^ in males with persistent AF aged ≥ 60 years, *p* < 0.0001). In younger patients, sex did not affect the extent of FAM (Supplemental Fig. 3, *p* = 0.389).

### Clinical risk stratification for fibrotic atrial myopathy and outcome-prediction in pulmonary vein isolation: the AF-SCORE

**Fig. 5:**
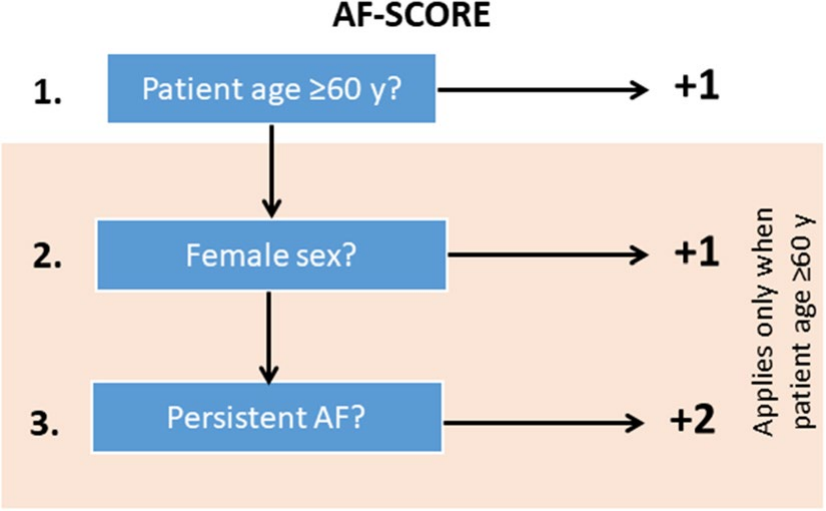
Determination of AF-SCORE. AF-SCORE is determined using patient age, sex, and clinical phenotype of AF. Patient age ≥ 60 years constitutes 1 point. Female sex (+ 1 point) and persistency of AF (+ 2 points) are added in patients aged ≥ 60 years. AF, atrial fibrillation

Based on multivariate logistic models (Table 1), age, sex and AF-phenotype were incorporated in a simple risk stratification scheme called AF-SCORE that ranges from 0 to 4 points (Fig. 5). One point is given when a patient’s age is ≥ 60 years, with additional points in older patients for female sex (1 point) and persistent AF-phenotype (2 points).

Forty-nine percent of patients in the mapping-validation cohort had an AF-SCORE ≤ 2. For “rule-out” of significant LVS, this threshold yielded a sensitivity of 92.0% and a negative-predictive value of 95.3%. A pathological threshold for AF-SCORE of ≥ 3 selected 51.4% of patients in the validation cohort and yielded a specificity of 64.9% and a positive-predictive value of 51.3% to “rule-in” significant LVS. Increasing the pathological threshold for AF-SCORE to 4 selected 15.9% of study patients and yielded a specificity of 93.6% and an increased positive-predictive value of 71.4% for “rule-in”.

**Fig. 6:**
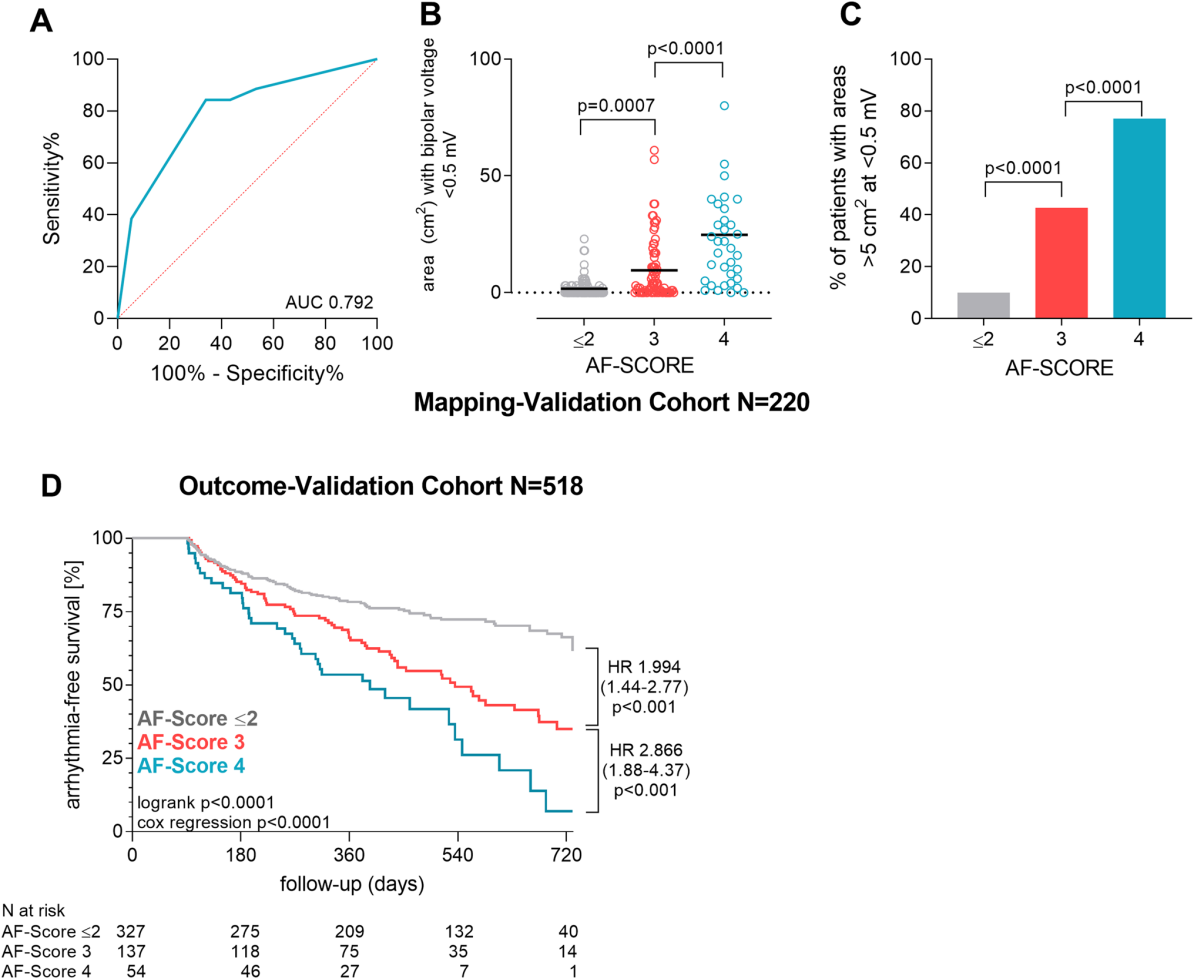
Diagnostic and prognostic properties of AF-SCORE. Receiver-operating-curve analysis of AF-SCORE for presence of FAM (≥ 5 cm^2^ low-voltage areas at < 0.5 mV) in the derivation cohort is given in (**A**). The absolute cumulative area with voltages < 0.5 mV with regard to AF-SCORE is given in (**B**). The percentage of patients with FAM, defined as cumulative areas with bipolar voltages < 0.5 mV of > 5 cm^2^ in relation to AF-SCORE, is shown in (**C**). Kaplan–Meier-estimates of arrhythmia-free survival following pulmonary vein isolation without additional left- or right-atrial ablations depending on AF-SCORE in the outcome-validation cohort is shown in (**D**). AUC, area under curve, HR, hazard ratio

Application of AF-SCORE to the mapping-validation cohort is shown in Fig. 6A–C. Receiver-operating-curve-analysis yielded an area-under-the-curve (c-statistic) for presence of FAM of 0.792 (Fig. 6A). An AF-SCORE of ≤ 2 is related to absence of, respective very minor FAM, which increases with an incremental AF-SCORE (Fig. 6B; 1.7 ± 3.9 cm^2^ for an AF-SCORE of ≤ 2, 9.6 ± 13.5 cm^2^ for an AF-SCORE of 3, and 24.8 ± 28.2 cm^2^ for an AF-SCORE of 4, *p* < 0.0001). Accordingly, the proportion of patients with FAM (> 5 cm^2^ at < 0.5 mV) increased incrementally from AF-SCORE ≤ 2 to AF-SCORE of 4 (Fig. 6C).

AF-SCORE as determined above was next applied on the outcome-validation cohort comprising 518 patients in total who underwent proximal-circumferential pulmonary vein isolation for symptomatic AF. Baseline characteristics of these patients with regard to outcome are given in supplemental table 2 and with regard to AF-SCORE in supplemental table 3. Patients with recurrence were older, predominantly female, had more often persistent AF, were more frequently ablated using radiofrequency than cryo-ablation and had a higher AF-SCORE. During a mean follow-up of 421 ± 208 days, 172 patients experienced AF-recurrence.

Patients with AF-SCORE of 3 or 4 demonstrated significantly more recurrences as opposed to patients with AF-SCORE 2 or lower (unadjusted hazard ratio 1.994 [95% confidence interval 1.44–2.77] for AF-SCORE 3 and HR 2.866 [95% CI 1.89–4.37] for AF-SCORE 4 compared to AF-SCORE ≤ 2; Fig. 6D). These differences persisted when the analysis was adjusted for type of ablation (HR 1.595 [95% CI 1.1–2.34] for AF-SCORE 3 and HR 2.231 [95% CI 1.39–3.59] for AF-SCORE 4 compared to AF-SCORE ≤ 2) or pre-procedural antiarrhythmic medications (HR 2.081 [95% CI 1.46–2.96] for AF-SCORE 3 and HR 3.443 [95% CI 2.22–5.33] for AF-SCORE 4 compared to AF-SCORE ≤ 2. Arrhythmia freedom rates were 74.3% (AF-SCORE ≤ 2), 54.7% (AF-SCORE 3) and 45.5% (AF-SCORE 4).

## Discussion

Our study reveals three major findings concerning the development of fibrotic atrial myopathy (FAM) in atrial fibrillation: first, age is the main factor in the development of FAM, and FAM is rare in those younger than 60 years. Second, sex and clinical phenotype of atrial fibrillation (AF) are important determinants with advanced age, but can de neglected in young patients. And third, incorporation of patient’s age, sex and AF-phenotype into AF-SCORE yields a simple, widely applicable tool to approximate the individual risk for FAM and to estimate the success rate of pulmonary vein isolation in both paroxysmal and persistent AF.

### Clinical risk scores for fibrotic atrial myopathy

Fibrotic remodeling of previously healthy atrial myocardium is associated with slow-conduction sites that serve as arrhythmogenic substrate and contribute to the perpetuation of AF even in the absence of pulmonary vein-inputs [3, 5, 9]. Fibrotic regions can be identified either as areas with reduced local voltage by endocardial contact mapping or by delayed Gadolinium-enhanced areas using MRI [10]. No matter the diagnostic modality, advanced fibrotic remodeling was demonstrated to be a main factor determining the long-term success of PVI [5, 6, 8, 11, 12]. In general practice, the use of these tools is, however, limited by prohibitive costs (MRI), invasiveness (contact mapping) and/or required expertise (electrocardiographic markers) [8, 12].

These limitations led to the development of several scoring systems that aim at stratifying patients for the presence of fibrotic atrial remodeling such as the DR-FLASH and APPLE-score [13, 14]. With an area-under-the-curve of 0.711 (APPLE-score) and 0.797 (DR-FLASH), they offer moderate to good discriminative properties for the diagnosis of atrial low-voltage substrate, but require echocardiographic or laboratory parameters such as left atrial diameter, left-ventricular ejection fraction or renal function in addition to routine patients’ clinical characteristics. In contrast, AF-SCORE as described in the current study relies solely on patient characteristics that are part of every AF-patient’s medical history: age, sex, and whether AF is paroxysmal or persistent. This greatly condensed clinical risk stratification model yields diagnostic properties (AUC 0.792) that are on par with the abovementioned scores and others, and AF-SCORE proved equally efficient to predict PVI-outcome.

### Atrial fibrosis in the aging heart

The current study underlines that age is a major determinant for the development of extra-pulmonary vein arrhythmogenic fibrotic substrate in the left atrium. This finding spreads new light on the interpretation of established clinical trials on AF-ablation, particularly on those that focus on persistent forms of AF: the STAR-AF II-trial compared PVI-only to complex fractionated atrial electrogram (CFAE)-ablation or linear ablation in addition to PVI, and found no benefit of adjunctive left atrial ablations [15]. This trial enrolled younger patients at a mean age of 58–61 years. Similar findings were obtained in the CHASE-AF trial, which compared a stepwise approach including widespread bi-atrial ablations (“full-defragmentation”) to PVI-only in persistent-AF patients [16]. At a mean age of 61 in the “full-defrag”-group, no additional benefit to PVI-only was observed.

Taking the current study’s data into account, a large proportion of patients in STAR-AF II and CHASE-AF likely had no relevant left-atrial arrhythmogenic fibrotic substrate, but rather isolated pulmonary vein-dependent AF – no matter of the clinical phenotype of persistent AF.

As a result, ablation strategies aiming at non-pulmonary vein-sources of AF such as linear ablations and ablation of high-voltage complex-fractionated atrial electrograms were in retrospect unlikely to show an additional benefit in these trials [15].

### Atrial fibrosis in the female heart

In patients aged 60 years or older, the current study found extensive non-pulmonary vein fibrotic substrate predominantly in females. However, women of any age are widely underrepresented in key clinical trials on AF-ablation: STAR-AF II and CHASE-AF included approximately 80% of male participants, and 87% of patients randomized to ablation in CASTLE-AF (comparing ablation to conservative medical therapy in patients with atrial fibrillation and heart failure) were males [17]. Even so, women in CASTLE-AF had no discernable benefit of catheter ablation in contrast to men in subgroup analysis.

Female sex seems disadvantageous with regard to fibrotic myocardial remodeling in at least two aspects. First, women, but not men, develop myocardial fibrosis with age also in the absence of cardiovascular risk factors [18]. And second, in specific cardiovascular disease such as valvular cardiomyopathy and despite a more beneficial overall cardiovascular risk profile, women have a higher burden of myocardial fibrosis than men [19].

### PV-dependent and non-PV-substrate-dependent persistent atrial fibrillation in contemporary clinical trials

Guidelines define persistent AF as individual AF-episodes that vary in duration from seven days to less than 12 months. The current study illustrates that this population is not homogeneous, but composed of two very different groups of patients: younger patients, in whom AF is largely PV-dependent, no matter the clinical AF-phenotype or sex, and older patients, particularly women (74% of females ≥ 60 years with persistent AF vs. 55% of males had FAM), who likely have fibrotic atrial substrate resulting in a “non-PV-substrate-dependent” form of persistent AF. Unfortunately, many key clinical trials on AF-ablation in general and ablation of persistent AF in particular were conducted in patient populations consisting of relatively young, male, and presumably PV-dependent AF-patients (Supplemental Fig. 4). Future studies investigating adjunctive left-atrial ablations in addition to PVI should therefore focus on patients with the highest likelihood of non-PV-substrate-dependency: senior patients and women with persistent AF.

## Limitations

The current study uses endocardial contact mapping to quantify FAM, which is routinely used for this purpose and can be considered the clinical standard. However, atrial histology would constitute the gold standard to quantify the fibrotic remodeling in FAM, but is unavailable in clinical practice.

Also, AF-SCORE was determined in a derivation cohort (supplemental Fig. 1) and then validated for FAM-extent and PVI-outcome in two separate, external validation cohorts (the so-called mapping-validation cohort and outcome-validation cohort; Fig. 6A–C and D, respectively). As not all patients in the outcome-validation cohort underwent high-density mapping, a direct link of FAM-extent to PVI-outcome can however not be extrapolated from the current study’s data.

## Conclusions

AF-SCORE stratifies atrial fibrillation-patients for FAM and risk for arrhythmia recurrences with clinically relevant diagnostic reliability. With expected arrhythmia freedom rates within two years from ablation approaching 75%, paroxysmal AF-patients of any age and younger patients with persistent AF are, irrespective of sex, promising candidates for PVI-only-techniques using either radiofrequency-ablation or single shot-cryo ablation. Challenges do remain in older patients with persistent AF, and particularly older women, in whom arrhythmia freedom can be achieved in only in half of patients using current PVI-only approaches.

## Supplementary Information

Below is the link to the electronic supplementary material.

Supplementary file1 (PDF 1518 kb)

## Data Availability

Data will be made available upon reasonable request.
