# Supplementary material for: Determinants of fibrotic atrial cardiomyopathy in atrial fibrillation. A multicenter observational study of the RETAC (reseau européen de traîtement d’arrhythmies cardiaques)-group
Source: Clin Res Cardiol. 2021 Dec 2;111(9):1018–27. doi: 10.1007/s00392-021-01973-1 (PMC9424172; doi:10.1007/s00392-021-01973-1)
Supplement: Supplementary file 1 — Supplementary file1 (PDF 1518 kb) [file 392_2021_1973_MOESM1_ESM.pdf]

SUPPLEMENTAL FIGURE 1

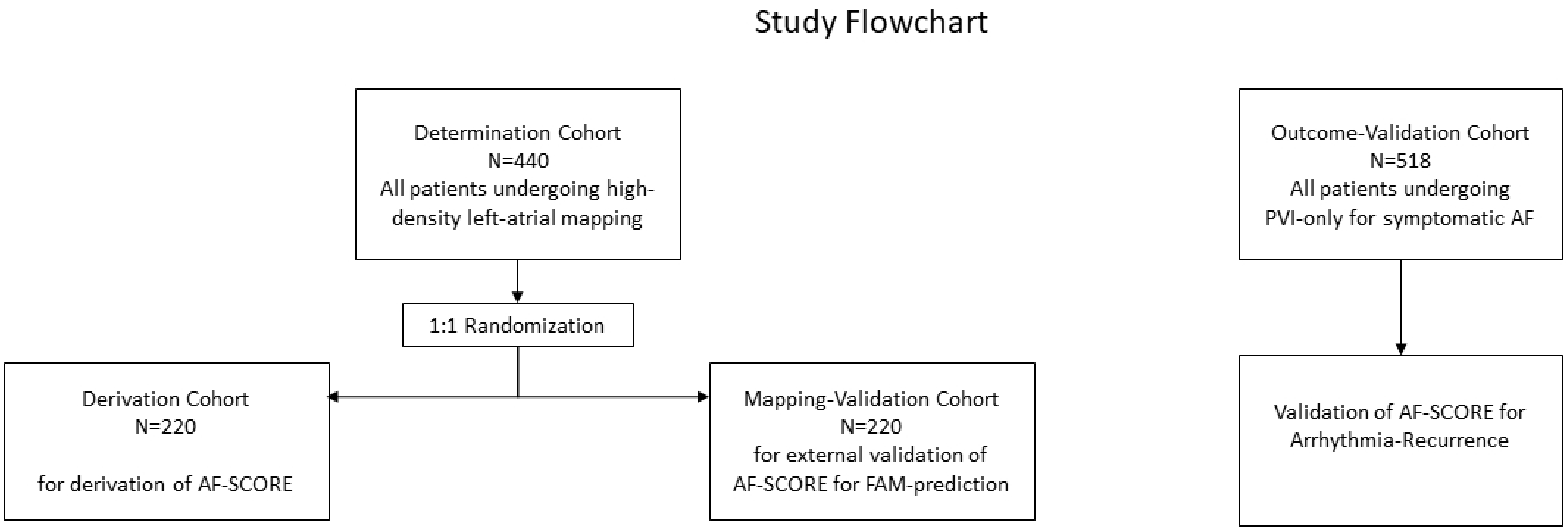

SUPPLEMENTAL FIGURE 2

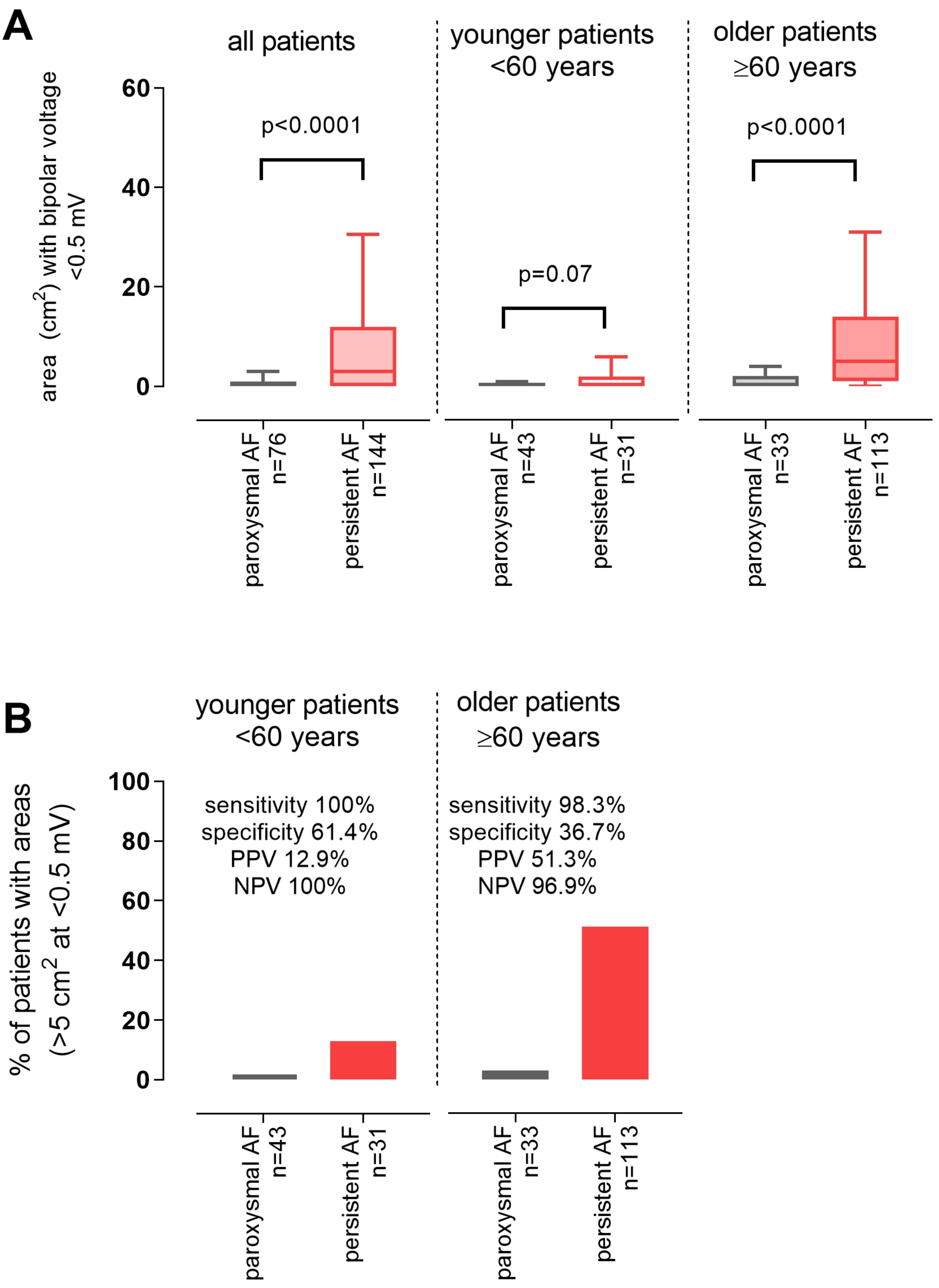

SUPPLEMENTAL FIGURE 3

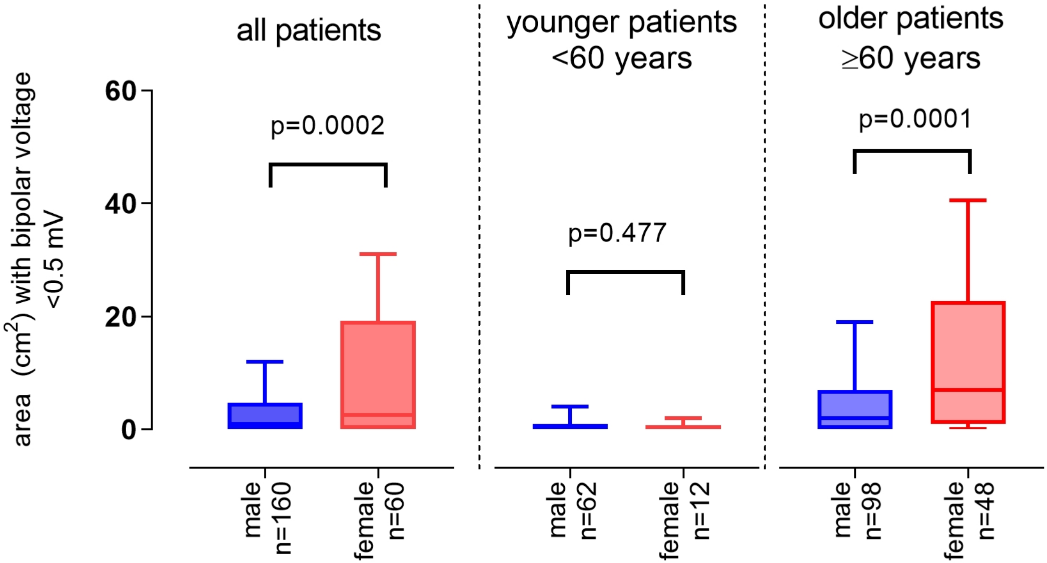

Supplemental Figure 4

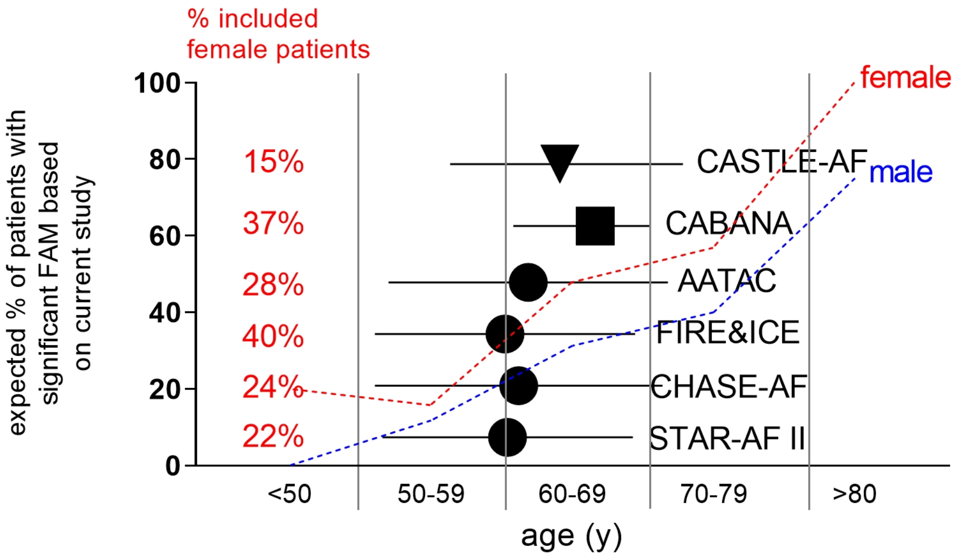

SUPPLEMENTAL TABLE 1: Comparison of Derivation- and Mapping-Validation Cohorts

	Derivation-Cohort n=220	Mapping-Validation Cohort n=220	p
Age (years)	61.1 (11.7)	62.8 (11.1)	0.112
Age >60 years	146 (66.4)	142 (64.5)	0.764
Female sex	60 (27.3)	64 (29.1)	0.751
Body mass index (kg/sqm)	28.0 (4.3)	27.7 (4.4)	0.460
Arterial hypertension	139 (63.2)	137 (62.3)	0.921
Diabetes mellitus	19 (8.6)	24 (10.9	0.521
Coronary artery disease	37 (16.8)	30 (13.6)	0.426
Persistent atrial fibrillation	144 (65.5)	157 (71.4)	0.218
LV dysfunction (LVEF<45%)	17 (7.7)	4 (1.8)	0.006
LVEDD (mm)	51.1 (4.4)	51.0 (4.3)	0.798
LA dilatation (>40 mm)	173 (78.6)	186 (84.5)	0.140
Creatinine clearance (ml/min/1.73 sqm)	78.3 (15.1)	80.0 (17.8)	0.284
Cumulative area <0.5 mV (sqcm)	6.1 (11.9)	8.1 (16.1)	0.138
cumulative area <1.0 mV (sqcm)	15.2 (21.1)	18.8 (27.6)	0.131

FAM, fibrotic atrial myopathy (prespecified as ≥5 cm2 with bipolar voltage <0.5 mV); LA, left atrial; LV, left ventricle; LVEDD, left-ventricular end-diastolic diameter

Values are given as mean±standard deviation or n (%)

SUPPLEMENTAL TABLE 2: Baseline characteristics of patients of the outcome-validation cohort

	**all patients** n=518	**no recurrence** n=341	**recurrence** n=177	*p*
Age	62.2 (10.1)	61.0 (10.2)	64.5 (9.3)	<0.0001
Female sex	139 (26.6)	81 (23.5)	58 (32.8)	0.028
Persistent atrial fibrillation	285 (54.6)	164 (47.7)	120 (67.8)	<0.0001
Ablation type				<0.0001
- Cryo	341 (65.3)	248 (72.1)	93 (52.5)	
- Radiofrequency	181 (34.7)	96 (27.9)	84 (47.5)	
Structural cardiomyopathy	79 (15.1)	54 (15.7)	25 (14.1)	0.696
Arterial hypertension	195 (43)	125 (41.7)	70 (39.5)	0.423
Left ventricular dysfunction (LVEF<45%)	44 (8-4)	25 (7.3)	19 (10.7)	0.181
Left atrial diameter	39 (12)	38 (11)	40 (12)	0.251
Pre-procedural antiarrhythmic drugs				0.305
- none	62 (12.1)	42 (12.6)	20 (11.3)	
- amiodarone	248 (48.5)	151 (45.2)	97 (54.8)	
- flecainide	111 (21.7)	79 (23.7)	32 (18.1)	
- sotalol	46 (9.0)	33 (9.9)	13 (7.3)	
- other	44 (8.6)	8.7)	15 (8.5)	
AF-SCORE	1.60 (1.45)	1.38 (1.38)	2.04 (1.5)	<0.0001

LVEF, left ventricular ejection fraction
Values are given as mean±standard deviation or n (%)

SUPPLEMENTAL TABLE 3: Baseline characteristics of patients of the outcome-validation cohort according to AF-SCORE

		AF-SCORE				
	all patients	**0**	**1**	**2**	**3**	**4**
n	518 (100)	186 (35.9)	82 (15.8)	59 (11.4)	137 (26.4)	54 (10.4)
Age	62.2 (10.1)	51.1 (7.1)	67.4 (5.5)	67.8 (5.1)	68.0 (5.0)	70.0 (5.6)
Female sex	139 (26.6)	25 (13.4)	0 (0)	59 (100)	0 (0)	54 (100)
Persistent atrial fibrillation	285 (54.6)	90 (48.4)	0 (0)	0 (0)	137 (100)	54 (100)
Structural cardiomyopathy	79 (15.1)	29 (16.9)	18 (22.0)	9 (15.3)	19 (19.6)	4 (7.4)
Arterial hypertension	195 (43)	61 (35.5)	35 (42.7)	21 (35.6)	52 (38.0)	26 (48.1)
Left ventricular dysfunction (LVEF<45%)	44 (8.4)	19 (11.0)	7 (8.5)	1 (1.7)	12 (8.8)	5 (11.6)
Left atrial diameter	39 (12)	38 812)	34 (14)	38 (13)	43 (8)	41 (8)

LVEF, left ventricular ejection fraction
Values are given as mean±standard deviation or n (%)

Supplemental Figure 1: Study Flowchart

Supplemental Figure 2: Clinical phenotype of atrial fibrillation and low-voltage substrate

The cumulative area with bipolar voltages <0.5 mV with respect to the clinical phenotype of AF in all patients of the derivation cohort (left panel), patients younger 60 years only (middle panel), and patients 60 years or older (right panel) is given in (**A**). The diagnostic performance of AF-phenotype to predict significant low-voltage substrate (>5 cm^2 at <0.5 mV) in younger (left panel) and patients aged 60 years or more (right panel) is shown in (**B**).

AF, atrial fibrillation; NPV, negative-predictive value; PPV, positive-predictive value

Supplemental Figure 3: Impact of sex on low-voltage substrate

Comparison of females and males in pooled data of any age (left panel), younger patients only (middle panel) and patients aged 60 years or more (right panel).

Supplemental Figure 4: Study Overview of Contemporary Clinical Trials on Atrial Fibrillation-Ablation

Shown is the reported age (in circles: mean and standard deviation; in squares: median and interquartile range; in triangles: median and range) of study patients of selected key clinical trials. The red and blue dashed lines visualize the percentage of patients that can be expected to have significant FAM (defined as >5 cm^2 of area with bipolar voltages <0.5 mV) based on the current study. On the left hand side in red is given the reported percentage of female

patients that were included in the given trials. For CHASE-AF e.g., only 24% of included patients were female. At a mean age of 62 years with a large male predominance, around 20% of patients in this trial likely had significant FAM.

FAM, fibrotic atrial myopathy

Supplemental Table 1: Comparison of Derivation- and Mapping-Validation Cohorts

Supplemental Table 2: Baseline characteristics of patients of the outcome-validation cohort

Supplemental Table 3: Baseline characteristics of patients of the outcome-validation cohort according to AF-SCORE
